# Biosynthesis and characterization of a recombinant eukaryotic allophycocyanin using prokaryotic accessory enzymes

**DOI:** 10.1002/mbo3.989

**Published:** 2020-01-22

**Authors:** Jorge Dagnino‐Leone, Maximiliano Figueroa, Elena Uribe, María Victoria Hinrichs, Diego Ortiz‐López, José Martínez‐Oyanedel, Marta Bunster

**Affiliations:** ^1^ Departamento de Bioquímica y Biología Molecular Universidad de Concepción Concepción Chile

**Keywords:** *Agarophyton chilensis*, functional characterization, recombinant allophycocyanin, structural characterization

## Abstract

Phycobiliproteins (PBPs) are colored fluorescent proteins present in cyanobacteria, red alga, and cryptophyta. These proteins have many potential uses in biotechnology going from food colorants to medical applications. Allophycocyanin, the simplest PBP, is a heterodimer of αβ subunits that oligomerizes as a trimer (αβ)_3_. Each subunit contains a phycocyanobilin, bound to a cysteine residue, which is responsible for its spectroscopic properties. In this article, we are reporting the expression of recombinant allophycocyanin (rAPC) from the eukaryotic red algae *Agarophyton chilensis* in *Escherichia coli*, using prokaryotic accessory enzymes to obtain a fully functional rAPC. Three duet vectors were used to include coding sequences of α and β subunits from *A. chilensis* and accessorial enzymes (heterodimeric lyase cpc S/U, heme oxygenase 1, phycocyanobilin oxidoreductase) from cyanobacteria *Arthrospira maxima*. rAPC was purified using several chromatographic steps. The characterization of the pure rAPC indicates very similar spectroscopic properties, λ_max_^Abs^, λ_max_^Em^, fluorescence lifetime, and chromophorylation degree, with native allophycocyanin (nAPC) from *A. chilensis.* This method, to produce high‐quality recombinant allophycocyanin, can be used to express and characterize other macroalga phycobiliproteins, to be used for biotechnological or biomedical purposes.

## Graphical abstract

A heterologous expression of Allophycocyanin from A.chilensis in E.coli was successfully accomplished. The enzymes for the synthesis of the holo‐APC were obtained from Arthrospira maxima, and they were active to introduce phycocyanobilin to Allophycocyanin. The spectroscopic and biochemical characterization of the protein confirmed a trimeric recombinant Allophycocyanin. The trimeric recombinant APC produced was completely functional, with high quality and it showed similar properties with native APC.

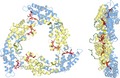

**Abbreviations**
ApcA: α subunit of allophycocyanin
ApcB: β subunit of allophycocyanin
*cpcS*/*cpcU*: genes for the subunits of the heterodimeric lyase
*ho1*: heme oxygenase gene
nAPC: native allophycocyanin of *Agarophyton chilensis*
PB: phycobilin
*pcyA*: phycocyanobilin oxidoreductase gene
rAPC: recombinant allophycocyanin of *Agarophyton chilensis*

## 1 INTRODUCTION

Phycobiliproteins are colored and highly fluorescent proteins that form the phycobilisome of algae, cyanobacteria, and cryptophyta. These proteins have been used as fluorescent markers (Patel, Mishra, Pawar, & Ghosh, 2005) and also for photodynamic therapy of cancer for their antioxidant, anti‐inflammatory, and antitumor properties (Li et al., 2019; Pagels, Guedes, Amaro, Kijjoa, & Vasconcelos, 2019).

Allophycocyanin (APC) is the main component of the phycobilisome core. APC is a heterodimer of αβ subunits. Each subunit contains a phycocyanobilin molecule synthesized by heme oxygenase 1 and phycocyanobilin oxidoreductase enzymes (Frankenberg‐Dinkel & Terry, 2009), bound to cysteine 82 by a heterodimeric lyase S/U (Schluchter, 2010). This pathway has been reported only for cyanobacteria. The subunits oligomerize as a trimers (αβ)_3_. The trimeric form of APC is the biologically functional state. This oligomeric state is necessary to provide the specific conformation and relative location of the chromophores to present the typical absorption and emission spectra of APC with λ_max_^Abs^ = 651nm and λ_max_^Em^ = 660 nm (MacColl, 2004; Samsonoff & MacColl, 2001). For its spectroscopic characteristic, purified APC is an important candidate for the biotechnological, pharmaceutical, cosmeceutical (Li et al., 2019; Pagels et al., 2019), and food industry (Dumay, Morançais, Munier, Le Guillard, & Fleurence, 2014). The recovery of allophycocyanin and other phycobiliproteins from natural sources requires large‐scale cultures of cyanobacteria or considerable amount of eukaryotic algae to be processed. Nevertheless, the production of recombinant phycobiliproteins in *E. coli* would reduce the costs and time required to obtain them with the necessary quality for biotechnological purposes. In the last decade, different protocols to obtain recombinant phycobiliproteins have been published in order to obtain molecular species with properties similar to the proteins purified from native organisms (Biswas, 2010; Liu et al., 2010). The production of α phycocyanin subunits with λ_max_^Abs^ at 625 nm and λ_max_^Em^ at 641 nm (Tooley & Glazer, 2002), subunits of αAPC (Hu, Lee, Lin, Chiueh, & Lyu, 2006; Liu et al., 2009), βAPC subunits of *Synechocystis sp* PCC6803 with λ_max_^Abs^ at 611 nm and λ_max_^Em^ at 642 nm (Chen, Lin, Li, Jiang, & Qin, 2013) are examples of these attempts. It has been reported also the obtaining of trimeric rAPC of *Synechocystis sp* PCC6803 in *E.coli*, by using multiple duet vectors (Liu et al., 2010) as well as from *Synechococcus* in *E. coli* (Biswas, 2010). These vectors contained the sequences for the subunits α and β of the phycobiliproteins, the enzymes to produce phycocyanobilin and the subunits of a heterodimeric lyase for the covalent binding of the chromophore to the corresponding cysteine. It has been reported that the isolated heterodimer (αβ) of a rAPC of *Synechocystis* sp PCC 6,803 obtained in *E.coli* has an absorption máximum at 615 nm, but when rAPC recovers its trimeric state (αβ)_3_, also recovers its absorption maximum at 650 nm (Liu et al., 2010). There is not enough information on the metabolic pathway for the synthesis and binding of the phycobilin (PB) to the holo‐APC in eukaryotic red alga. The whole genome of *P. cruentum* (Bhattacharva et al., 2013) and the plastid genome and transcriptome of *A. chilensis* (Hagopian, Silva Reis, & Kitayima, 2004; Vorphal et al., 2017) have been reported, but it was not possible to find the sequences of the enzymes of the pathway. In this article, we modify the methodology by using heterologous enzymes, to improve the chromophorylation of the trimers (αβ)_3_ we used three duet expression vectors and a His‐tag only in the β subunits to avoid steric hindrance.

Our group previously had studied APC of *Gracilaria chilensis* (Dagnino‐Leone, 2017), from now on called *Agarophyton chilensis* (Le, Fredericq, Norris, Gurgel, & Schmidt, 2018). According to this information, native APC (nAPC) present in *A. chilensis* is trimeric. In eukaryotic red algae, APC is extracted in lower amount than the other phycobiliproteins because, as part of the core, it is associated to membranes through the linker core‐membrane (Li et al., 2016; Tang et al., 2015). For biotechnological purposes, it is necessary that the recombinant APC (rAPC) be in a single oligomerization state as a trimer, highly chromophorylated, with the correct spectroscopic properties, and with a high yield after the purification process. In this article, we present an approach to obtain a trimeric allophycocyanin from a eukaryotic macroalgae in a prokaryotic system. To do that, three duet expression vectors were used, which contains coding sequences of *A. chilensis* allophycocyanin α and β subunits, and enzymes to obtain holo‐APC (*heterodimeric lyase cpc S/U, hemeoxygenase 1, phycocyanobilin oxido reductase*) from the cyanobacteria *Arthrospira maxima*. The expression was accomplished, and the protein rAPC was purified and compared with native allophycocyanin from *A. chilensis* (nAPC) by absorption and emission spectroscopy, circular dichroism, and molecular sieve chromatography. This method leads to the production of rAPC with very similar properties to nAPC, which can be used for biotechnological purposes.

## 2 MATERIALS AND METHODS

### 2.1 DNA extraction and PCR conditions

The coding sequences of the six genes needed were amplified by PCR using KAPA HiFi polymerase. *apcA* and *apcB* were obtained using *A. chilensis* DNA as template. The purification of DNA from *A. chilensis* was performed according to the literature (Ramakrishnan, Fathima, & Ramya, 2017). PCR were performed using the following primers: *apcAf*: 5ʹ‐CCATGGGTATTATTACTAAATCAATCGTTAAT‐3ʹ, *apcAr*: 5ʹ‐GGATCCTTACTGCATTGCACCTAATGT‐3ʹ, *apcBf*: 5ʹ‐GGA TCCAATGCAAGATGCTATTACTTCT‐3ʹ, *apcBr*: 5ʹ‐GAGCTC CTAGCTTAAACCAGAACAAAT‐3ʹ. The coding sequences of *pcyA (NZ ABYK01000007), ho1(NZ ABYK01000010), cpcU(NZ ABYK01000007), cpcS(NZ ABYK01000019)* from *A. maxima* were obtained from GenBank. The purification of total DNA from *A. maxima* (strain donated by Prof. Mariela González from Facultad de Ciencias Naturales, Universidad de Concepción) was performed according to the literature (Morin, Vallaeys, Hendrickx, Natalie, & Wilmotte, 2010). PCR were performed using the following primers: *cpcSf*; 5ʹ‐CATATGATGGATGCTATAGAATTTTT‐3ʹ, *cpcSr*: 5ʹ‐CTCGAGTTACCAACCAAAGGC‐3ʹ, *cpcUf*; 5ʹ‐CATATGATGGATATT GTCGAA‐3ʹ, *cpcUr*: 5ʹ‐CTCGAGTTACTTTAAACCCAT‐3ʹ: 5ʹ‐CATATGATGGATATTGTCGAA‐3ʹ, *pcyAr *5ʹ‐CCATGGATGCAATCA ACTTAC‐3ʹ, *hoI*f, 5ʹ‐CCATGGATGAGTGTTAATCTAG –*hoIr*: 5ʹ‐GGATCCTTTCATGTTCATTCC‐3ʹ. In bold are shown the restriction sites for subcloning.

Each gene was cloned in TOPO‐TA 2–1 vector (Thermo Fisher Scientific) and transformed into *E.coli* DH5α. Plasmid DNA was extracted with GeneJet mini plasmid kit (Thermo Fisher Scientific).

### 2.2 Construction of the expression vectors

Expression vectors were constructed as follows: *apcB* and *cpcU* in their cloning vectors were digested with *BamHI* and *SacI* for *apcB* and *NdeI* and *XhoI* for *cpcU* and then ligated at the cloning site 1 and 2 in pETDuet‐1 vector (novagen), respectively. *apcA* and cpcS were digested with *NcoI* and *BamHI,* and cpcS with *NdeI* and *XhoI* to be then ligated in the cloning site 1 and 2 of pCDFDuet‐1 vector (Novagen), respectively. Finally, *pcyA* and *ho1* were digested with *NcoI* and *BamHI* and *hoI* with *NdeI* and *xhoI*, they were cloned in pRSFDuet‐1 cloning sites 1 and 2 of, respectively. The expression vectors were sequenced at the Department of Ecology from Pontificia Universidad Católica de Chile and analyzed with Bioedit software to confirm the absence of mutations.

### 2.3 In vivo heterologous expression of recombinant Allophycocyanin (rAPC)

30 ng of each expression vectors, pCDF‐apcA‐cpcS, pET‐apcB‐cpcU y pRSF‐pcyA‐ho1, were co‐transformed in electrocompetent *E coli* BL21 (DE3). A Bio‐Rad Micropulser electroporator was used with a 2.5 kV transformation protocol for five milliseconds using a 2‐mm gap cuvette. The transformed bacteria were grown in LB‐Agar plates supplemented with 15 mg/L kanamycin, 25 mg/L streptomycin, and 50 mg/L ampicillin. The plates were incubated for 16 hr at 37°C. Starter culture (SC) was prepared with one colony in 20 ml TB (Terrific broth) under stirring at 37°C for 16 hr. 5 ml of SC was added to 500 ml TB supplemented with the same concentration of antibiotics and incubated at 37°C under stirring until OD_600nm_ = 0.6. The expression of the proteins was induced with IPTG (isopropyl‐β‐D‐thiogalactoside) to a final concentration of 1.0 mM. The culture was incubated under stirring at 30°C for 4 hr. After separation of the cells at 5,000 x *g* during 5 min, the bacterial pellet was suspended in 20 ml lysis buffer (50 mM K_2_HPO_4_/KH_2_PO_4_ pH 7, 150 mM KCl, 10% glycerol, and 5 mM dithiothreitol) in presence of proteases inhibitors (cOmplete mini EDTA free, Roche) and then lysed by sonication in ice bath, for 5 min (10 s sonication, 30 s pause). The total lysate was centrifuged at 15,000 x *g* for 20 min. The supernatant was used as input for the following purification steps.

### 2.4 Purification of rAPC

The proteins were precipitated with ammonium sulfate (60% saturation) at 4°C, during 16 hr; the sample was then centrifuged for 20 min at 15.000*g*. The protein pellet was dissolved in 5 ml of Buffer A (50 mM K_2_HPO_4_/KH_2_PO_4_ pH 7) supplemented with 10% glycerol. After dialysis versus Buffer A, for 12 hr (D‐Tube Mega 3.5 MWCO [Merck]), the protein solution was loaded in a DEAE FF 16/10 (GE Life Science) column, equilibrated with Buffer A (Flux: 2 ml/min). For the elution, a lineal gradient from 15% to 85% 1 M KCl in Buffer A was used at the same flux. The fractions with absorption at 651 nm were pooled and injected in a 1 ml IMAC HiTrap TALON crude column and washed with 6 ml of Buffer A, before the elution with a linear gradient of 15–500 mM imidazole in Buffer A. The fractions with absorption at 651 nm were subsequently pooled and purified by molecular exclusion in a Superdex 200 HiLoad 16/60 (Amersham) column, equilibrated with Buffer A, at a flux of 0.5 ml/min. All the chromatographic procedures were performed in an AKTA Prime (GE) system. The purified rAPC was concentrated in Amicon Ultra‐15 50 K, to 0.5 mg/ml and stored at −20°C. In parallel, nAPC was purified as reported previously (Dagnino‐Leone, 2017) for comparative proposes.

### 2.5 Characterization of rAPC

Purified proteins were analyzed by native PAGE(not shown), denaturating SDS‐PAGE and Western blot, using the His‐tag on the β subunit for detection. Absorption and emission spectra of rAPC and nAPC were recorded in a Jasco V‐650 spectrometer and a SHIMADZU RF–5301 PC spectrofluorimeter. The samples with a ratio A651/A280 >4 and with emission at 660 nm upon excitation at 651 nm were used for the oligomeric characterization.

The oligomerization state of rAPC and nAPC was determined in a molecular sieve chromatography with a Superdex 200 HiLoad 16/60 column (Amersham). The oligomer size was estimated using a calibration curve of molecular standard. Three replica of MW standards from 1,750–670,000 Da (*Gel filtration standard*, Bio‐Rad) were performed.

Circular dichroism spectra (190–250 nm) of nAPC and rAPC were recorded in a Jasco J‐1500 spectropolarimeter with PM‐539 detector and Peltier PTC‐517 at the Centro de Estudios para el Desarrollo de la Química (CEPEDEQ), Universidad de Chile. For measurement of the thermal stability, the circular dichroism signal following the changes in ellipticity at 222 nm between 25 and 75°C (0.5°C/min) was recorded. The protein concentrations were 0.1 mg/ml in both experiments.

The fluorescence lifetime was measured in a Fluo Time 200 (PicoQuant Inc) fluorimeter, with diode Lasers and LEDs as excitation source and an ultrafast MCP detector at Molecular Physics Lab, Christian Texas University. The measurements were performed with an angle of 54.7°, and the data were adjusted with software FluoFit4, for multicomponent systems (*t*) = ∫(*t*′)*t*−∞Σ*Aini* = 1*e*−*t*−*t*`*τt* *dt*.

The chromophorylation degree was determined for nAPC and rAPC based on (Biswas, 2010; Glazer, 1988) in two independent experiments.

## 3 RESULTS

### 3.1 Construction of the expression system for rAPC

The sequences of the genes heme oxygenase 1 (*ho1*), phycocyanobilin oxido reductase (*pcyA*)*,* lyase S subunit (*cpcS*), and lyase U subunit (*cpcU*) of *A. chilensis* have not been identified yet. The similitude among proteins involved in the phycobilisome system, for example APC of *A. chilensis* and *A.maxima* (82% and 83% identity for the α and β subunit, respectively) or heme oxygenase 1 of Gracilariae sp and *A.maxima* (45% identity), suggested that the metabolic pathways for their synthesis could be also similar although *apcA* and *apcB* genes are located at the chloroplast in *A. chilensis* and in the genomic DNA in *A. maxima*. It was not possible to find sequences reported for eukaryotic phycocyanobilin oxide reductase or lyases in public databases. The genome of *A. maxima* (Xu et al., 2016) was available, so as a thoughtful alternative, the three necessary enzymes from *A. maxima* were used. All the expression vectors show no mutations in the coding sequences.

### 3.2 Purification of rAPC

The purification of rAPC was performed from a pellet of 3.94 g of recombinant bacteria. Fractions of the semipurified rAPC from the ionic exchange chromatography account for 7 mg/L of bacterial culture, following its absorption at 651 nm. The following chromatography step with IMAC was also followed at 651 nm; two turquoise fractions were identified, the fraction retained in the column and the flow‐through fraction. Both protein fractions were analyzed. Even though both fractions have a λ_max_^Abs^ close to 651 nm, only the retained fraction showed an identical spectrum with nAPC. This fraction accounts for 0.124 mg/L of bacterial culture. The characterization of this fraction is presented below.

### 3.3 Characterization of rAPC

**Figure 1:**
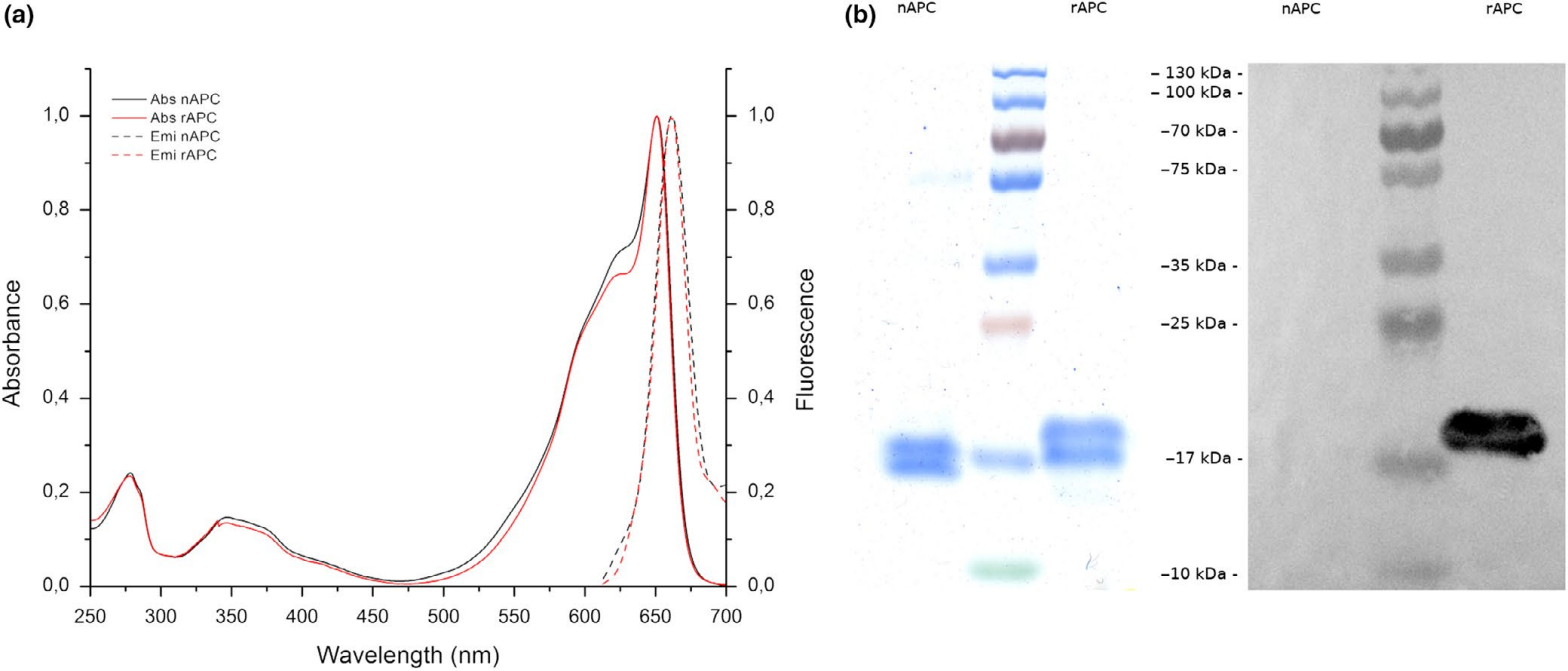
Spectroscopic and electrophoretic characterization of recombinant and native allophycocyanin. (a) Absorption and emission spectra of nAPC (red) and rAPC (black). (b) Electrophoretic characterization of rAPC and nAPC. Left: Coomassie blue‐stained SDS‐PAGE, Right: Western blot using antibody anti‐6XHis; this tag is only present in the β subunits

The purified rAPC showed the characteristic turquoise color. Its absorption and emission spectra are shown in Figure 1a. For both, nAPC and rAPC, their spectroscopic characteristics are very similar, with an absorption maximum at 651 nm, a shoulder at 620 nm and an emission maximum at 661 nm. Figure 1b shows the electrophoretic characterization of the purified protein (A651 nm/A280 nm >4). In SDS‐PAGE (Figure 1b), two bands at the estimated size of 17–19 kDa corresponding to α and β subunit of APC can be observed. The sample of rAPC presents a band of a slightly higher size. The Western blot shows signal only in the rAPC sample (Figure 1b).

**Figure 2:**
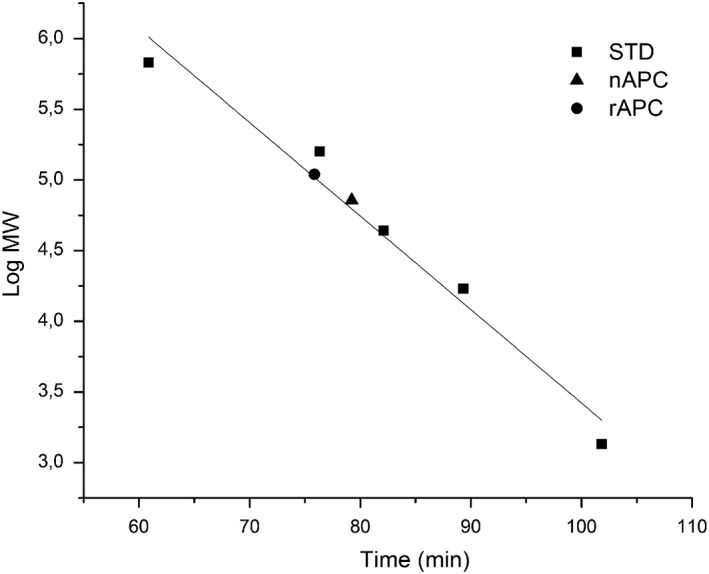
Molecular weight determination of functional rAPC. The molecular weight of the recombinant protein was determined by size exclusion chromatography, using a Superdex 200 16/60 column. The recombinant protein (rAPC) was compared with the native one (nAPC) obtaining retention time equivalents to 150 and 112 KDa, respectively. The Mw standards used for calibration are showed as well (STD)

The oligomerization state of nAPC and rAPC, determined by size exclusion chromatography, shows an estimated size of each protein, corresponding to MW of 112 kDa and 150 kDa, respectively (Figure 2).

**Figure 3:**
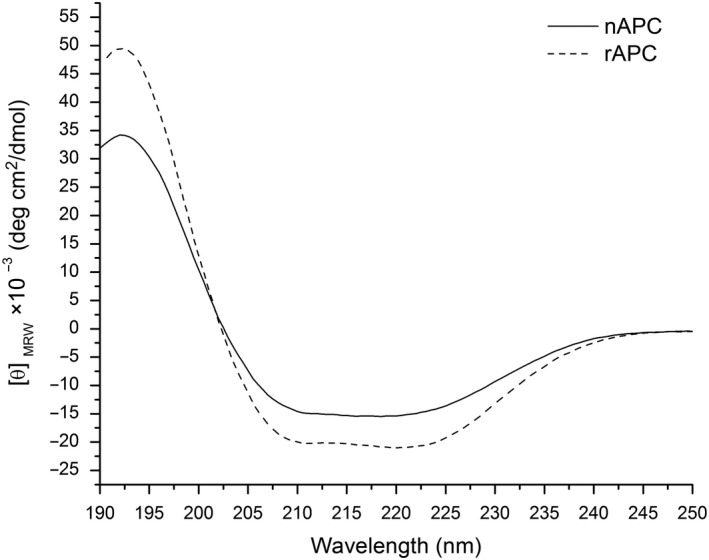
Circular dichroism spectra of nAPC and rAPC. Normalized Far‐UV CD spectra for both protein are shown. The spectra for both protein are very similar, with the features of proteins with mainly helices as secondary structure elements

**Figure 4:**
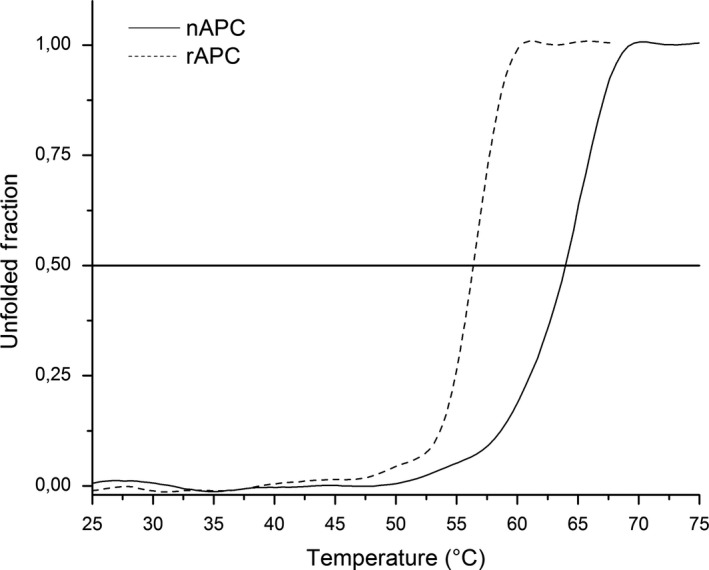
Thermal stability characterization of nAPC and rAPC. Normalized thermal denaturation plots, followed by CD ellipticity at 222 nm for both proteins, are shown. nAPC is more stable than rAPC protein, displaying Tm values of 64 and 56°C, respectively

The circular dichroism spectra for rAPC and nAPC are very similar showing the profile of predominant α helices as secondary structures, with minima at λ 208 and 222 nm and a maximum at 190 nm (Figure 3). The temperature of melting (Tm) determined by circular dichroism shows a Tm for nAPC of 64°C and a Tm of 56°C for the rAPC (Figure 4).

The fluorescent lifetime (τ) for each protein was measured, and the values are τ _nAPC_ = 1.65 ns and τ _rAPC_ = 1.64 ns indicating a completely functional protein.

**Figure A1:**
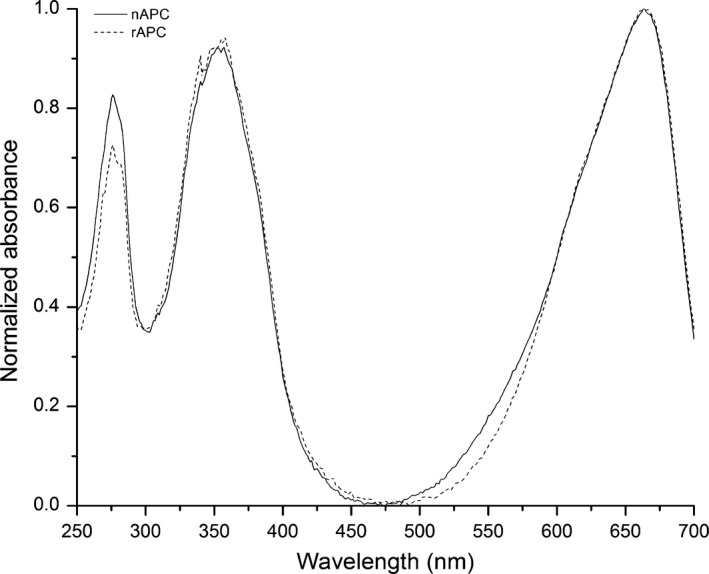
Spectra of denatured rAPC and nAPC for the determination of the chromophorylation efficiency. *The samples were denatured in PBS 50 mM, 8 M urea pH2 and the spectra were recorded. The method described by Biswas 2010 and Glazer 1988 used the molar extinction coefficient ε = 35.4 mM/cm or cyanobilins in absence of the protein environment (folded), and in this article, the molar extinction coefficient for folded rAPC and nAPC of 14.9mM/cm was used for the calculations.

The degree of chromophorylation was close to 50%, (nAPC: 52%, rAPC: 57%). Figure A1 shows the spectra for the denatured nAPC and rAPC that were used for the calculations. rAPC and nAPC showed similar degree of chromophorylation. Table 1 shows a comparison of the characteristics of rAPC and nAPC.

**Table 1:** Characteristics of nAPC and rAPC

	nAPC	rAPC
λ_max_^Abs^	651 nm Shoulder 620 nm	651 nm Shoulder 620 nm
λ_max_^Em^	662 nm	662 nm
τ	1.65 ns	1.64 ns
Oligomeric state	(αβ)_3_	(αβ)_3_
Tm	64°C	56°C
Chromophorylation degree	52%	57%

## 4 DISCUSSION

Phycobiliproteins have an enormous biotechnological potential, their applications go from food colorant to biomedical uses because they possess antioxidant and antitumorous properties. They are also used in photodynamic therapies as fluorescent probes because their spectroscopic characteristics. Allophycocyanin is the most simple phycobiliprotein, it possesses only one phycocyanobilin molecule per subunit attached to the peptide backbone, and its native functional oligomer is a trimer (De Marsac, 2003; MacColl, 1998, 2004). Phycobiliproteins from red algae are much less studied than cyanobacterial. An exception is the red microalgae *Porphyridium cruentum* (Bermejo, Ruiz, & Acien, 2007; Bermejo, Talavera, & Alvarez‐Pez, 2001; Nagy, Bishop, Klotz, Glazer, & Rapoport, 1985), but for eukaryotic macro algae the studies of phycobiliproteins are only a few (Galland‐Irmouli et al., 2000; Lüder, Knoetzel, & Wiencke, 2001). Allophycocyanin is the less abundant in eukaryotic phycobilisomes (Glazer, 1988) fact that presents a problem for the study and biophysical characterization of this protein.

**Figure 5:**
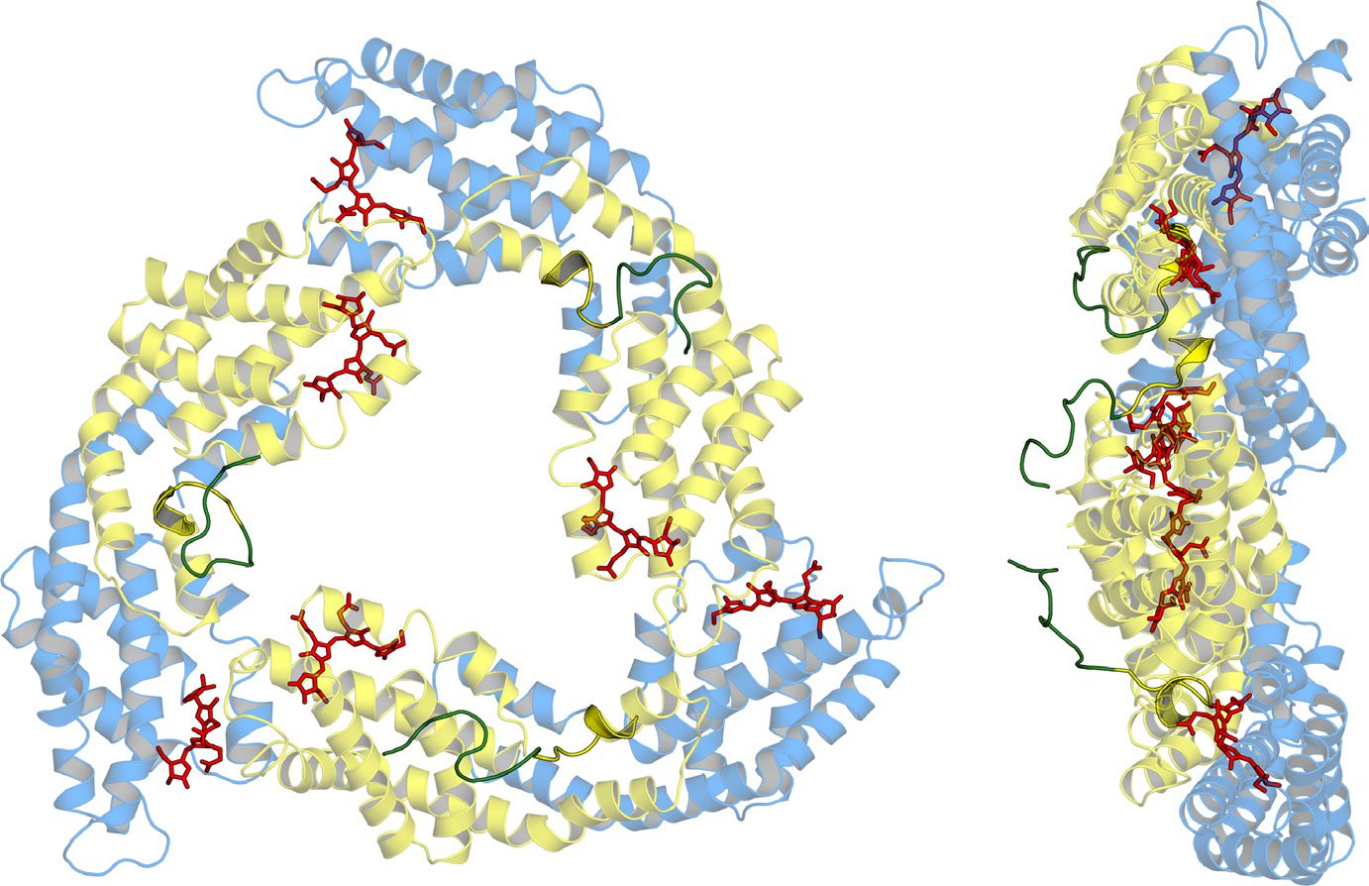
Molecular model of the structure of rAPC from *Agarophyton chilensis*. Yellow: α subunits, light blue: β subunits, red: the phycocyanobilins. Schematically, the position of the His‐tag is shown in green. On the left, frontal view, on the right a lateral view

In this work, we have obtained an eukaryotic recombinant allophycocyanin, rAPC, from *A. chilensis* using prokaryotic accessory enzymes (heterodimeric lyase S/U, hemeoxigenase 1 and phycocyanobilin oxido reductase) from *A. maxima* in *E.coli* with their spectroscopic and biochemical properties comparable to the purified native allophycocyanin. The expression system designed is based on the literature but with changes in order to obtain a fully functional protein. We designed an expression system to produce equivalent number of copies for α and β subunits. This was confirmed by the SDS‐PAGE (Figure 1) in which the intensity of the bands stained with Coomassie blue was also similar. The expression system was also selected to obtain a higher number of copies for the enzymes responsible for the synthesis and binding of the chromophores by using the vector pRSF which has a replication time 5 times faster than the vector that contained the α and β subunits of rAPC. This is important because the objective was to obtain a high degree of chromophorylation. Another important difference was the addition of a His‐tag only to the N‐terminal of β subunits, instead of the N‐terminal of α subunits (Biswas, 2010) or to the N‐terminal of both subunits as described in Liu et al., (2010). The structural information we had on APC (Dagnino‐Leone, 2017) was used in order to have less effect in the oligomerization state as a trimer. Figure 5 shows the molecular model of *A. chilensis* rAPC trimer, the position of the His‐tag is indicated and it was designed to eliminate the steric hindrance that could be produced in the organization of the subunits, and it would account for the trimeric oligomerization state obtained with this protocol. In previous reports ( Biswas, 2010), a different combination of three duet vectors were used*;* in (Liu et al., 2010) the authors included the six necessary genes in two expression vectors, inserting *cpcS* and *cpcU* in tandem in the cloning site 2 of pCDF vector and also *ho1* and *pcyA* in the cloning site 2 of vector pRSF. In both cases, lower chromophorylation efficiency (27%) was reported, compared with the expected for a native protein. In our case, we reached similar chromophorylation degree for the recombinant protein in comparison with the native one using the same methodology.

rAPC showed the characteristic spectrum of native APC, an absorption máximum at λ = 651 nm with a shoulder at λ = 620 nm, an emission máximum at λ = 660 nm, and very similar to those reported in literature (MacColl, 1983, 2004; MacColl, Csatorday, & Berns, 1981) for cyanobacterial APC.

Experimental information about the oligomeric state of nAPC in other red alga was not available even though in (Murakami, Mimuro, Ohki, & Fujita, 1981) the authors reported a trimeric state for the functional APC purified from *Anabaena cylindrica.* Our results from molecular sieve chromatography point to a trimeric state for nAPC from *A. chilensis* and for rAPC. This result agrees with the models obtained by X‐ray crystallography which report (αβ)_3_ as the biological unit (Dagnino‐Leone, 2017), (Brejc, Ficner, Huber, & Steinbacher, 1995; McGregor, Klartag, David, & Adir, 2008; Murray, Maghlaoui, & Barber, 2007; Schmidt, Krasselt, & Reuter, 2006). The circular dichroism spectrum for nAPC as well as for rAPC agrees with the secondary structure reported for the crystallographic structures of APC for *Agarophyton chilensis *(PDB ID: http://www.rcsb.org/pdb/search/structidSearch.do?structureId=5TJF; Bhattacharva et al., 2013), and also agrees with other APC structures from cyanobacteria reported at the PDB (Brejc et al., 1995; McGregor et al., 2008; Murakami et al., 1981; Murray et al., 2007), with predominance of helical structures revealed by the two minima at 222 and 208 nm. The deconvolution of the Far‐UV CD spectrum of rAPC reveals a 71% of helical content, in complete agreement with the 76% reported for the crystallographic structure of the nAPC considering that the His‐tag contributes to the spectra lowering the percentage of helical structures.

The main difference between nAPC and rAPC of *A. chilensis* is the melting temperature. The value of Tm for nAPC was 64°C and Tm for rAPC was 56°C. This difference could be assigned to the presence of the His‐tag used to facilitate the purification of the recombinant protein.

The His‐tag is associated at the N‐terminal of the *A.chilensis* allophycocyanin β subunit (Sequence added: MGSSHHHHHHSQDP), and there are three in each trimer. Considering that the analysis of the trimer showed a distance between tags of 40 Å; so it is possible that they could clash among them and its mobility would increase along with temperature, this fact would account for a decrease of 8°C in Tm for the rAPC as compared with nAPC. This behavior has been reported before (Khan, Legler, Mease, & Duncan, 2012) for native and recombinant proteins involving His‐tag for recognition. The presence of the tags also could explain the differences in the circular dichroism spectrum and the difference in the elution time during the molecular sieve chromatography in which the His‐tag should be responsible of the change in the hydrodynamic volume. Molecular models of the rAPC suggest this possibility.

The fluorescence lifetimes (τ) are also identical showing that rAPC and nAPC have similar functional properties. It has been reported that *Synechocystis sp*. PCC6803 also has also a similar value for τ (Maksimov et al., 2014).

The spectrum of nAPC and rAPC at denaturing conditions (8 M urea, pH 2) and the relationship between the concentrations of phycocyanobilin chromophore allows calculating the degree of chromophorylation of nAPC and rAPC. These values were the same with an estimated value of 52% and 57%. Biswas (2010) report that for monomers, the chromophorylation rate is 40% and for the trimer totally chromophorylated is 6.4%.

To this point, the *A. chilensis* rAPC showed very similar properties with nAPC, as it is shown on Table 1.

We were able to produce 7 mg/L of recombinant rAPC, but only 0.124 mg/L corresponds to a functional trimeric conformation. Biswas et al (10) reported the production of 5 to 12.4 mg of rAPC from *Synechococcus* sp. strain PCC 7,002, but they did not report the amount of functional protein for comparison (Biswas, 2010). More experiments are needed to fine tuning the protein expression of *A. chilensis* rAPC. Changing temperature and induction time would allow optimize the production of functional *A. chilensis* rAPC in *E. coli*.

In summary, we have obtained a recombinant eukaryotic allophycocyanin in its trimeric and functional conformation, by using a cyanobacterial enzymatic accessory system. rAPC has very similar properties with nAPC, and it is completely functional for biotechnological and/or biomedical purposes. In addition, this system would allow the study of the biophysical characteristics of the other subunits with different spectroscopic properties, present in the core of the phycobilisome of *Agarophyton chilensis*, such as α^II^ and β^18^. The system also will allow the production of other recombinant phycobiliproteins from other red macroalga for biotechnological purposes.

## CONFLICT OF INTEREST

None declared.

## AUTHOR CONTRIBUTION

Jorge Dagnino‐Leone: Conceptualization; Investigation; Methodology; Writing‐original draft. Maximiliano Figueroa: Conceptualization; Investigation; Methodology; Writing‐review & editing. Elena Uribe: Methodology; Resources. Maria Victoria Hinrichs: Investigation; Methodology. Diego Ortiz‐López: Formal analysis; Investigation; Methodology. José Martínez‐Oyanedel: Conceptualization; Data curation; Formal analysis; Methodology; Supervision; Validation; Writing‐original draft; Writing‐review & editing. Marta Bunster: Conceptualization; Formal analysis; Funding acquisition; Investigation; Methodology; Project administration; Resources; Supervision; Writing‐original draft; Writing‐review & editing.

## ETHICS STATEMENT

None required.

## Data Availability

All data generated or analyzed during this study are included in this published article.
